# Genome-Wide Association Identifies Multiple Genomic Regions Associated with Susceptibility to and Control of Ovine Lentivirus

**DOI:** 10.1371/journal.pone.0047829

**Published:** 2012-10-17

**Authors:** Stephen N. White, Michelle R. Mousel, Lynn M. Herrmann-Hoesing, James O. Reynolds, Kreg A. Leymaster, Holly L. Neibergs, Gregory S. Lewis, Donald P. Knowles

**Affiliations:** 1 Animal Disease Research Unit, Agricultural Research Service, U. S. Department of Agriculture, Pullman, Washington, United States of America; 2 Department of Veterinary Microbiology and Pathology, Washington State University, Pullman, Washington, United States of America; 3 U. S. Sheep Experiment Station, Agricultural Research Service, U. S. Department of Agriculture, Dubois, Indiana, United States of America; 4 Genetics and Breeding Research Unit, U. S. Meat Animal Research Center, Agricultural Research Service, U. S. Department of Agriculture, Clay Center, Nebraska, United States of America; 5 Department of Animal Sciences, Washington State University, Pullman, Washington, United States of America; Naval Research Laboratory, United States of America

## Abstract

**Background:**

Like human immunodeficiency virus (HIV), ovine lentivirus (OvLV) is macrophage-tropic and causes lifelong infection. OvLV infects one quarter of U.S. sheep and induces pneumonia and body condition wasting. There is no vaccine to prevent OvLV infection and no cost-effective treatment for infected animals. However, breed differences in prevalence and proviral concentration have indicated a genetic basis for susceptibility to OvLV. A recent study identified *TMEM154* variants in OvLV susceptibility. The objective here was to identify additional loci associated with odds and/or control of OvLV infection.

**Methodology/Principal Findings:**

This genome-wide association study (GWAS) included 964 sheep from Rambouillet, Polypay, and Columbia breeds with serological status and proviral concentration phenotypes. Analytic models accounted for breed and age, as well as genotype. This approach identified *TMEM154* (nominal P = 9.2×10^−7^; empirical P = 0.13), provided 12 additional genomic regions associated with odds of infection, and provided 13 regions associated with control of infection (all nominal P<1×10^−5^). Rapid decline of linkage disequilibrium with distance suggested many regions included few genes each. Genes in regions associated with odds of infection included *DPPA2*/*DPPA4* (empirical P = 0.006), and *SYTL3* (P = 0.051). Genes in regions associated with control of infection included a zinc finger cluster (*ZNF192*, *ZSCAN16*, *ZNF389*, and *ZNF165*; P = 0.001), *C19orf42*/*TMEM38A* (P = 0.047), and *DLGAP1* (P = 0.092).

**Conclusions/Significance:**

These associations provide targets for mutation discovery in sheep susceptibility to OvLV. Aside from *TMEM154*, these genes have not been associated previously with lentiviral infection in any species, to our knowledge. Further, data from other species suggest functional hypotheses for future testing of these genes in OvLV and other lentiviral infections. Specifically, *SYTL3* binds and may regulate *RAB27A*, which is required for enveloped virus assembly of human cytomegalovirus. Zinc finger transcription factors have been associated with positive selection for repression of retroviral replication. *DLGAP1* binds and may regulate *DLG1*, a known regulator of HIV infectivity.

## Introduction

Ovine lentivirus (OvLV), like the human immunodeficiency virus (HIV), is a macrophage-tropic lentivirus that leads to persistent, lifelong infection of the host [1], [2]. Seroprevalence of OvLV is 24–26% in U.S. domestic sheep, with positive animals in approximately half of all U.S. flocks [3], [4]. Ovine lentivirus infection leads to varying degrees of dyspnea (respiratory distress), cachexia (body condition wasting), mastitis, arthritis, and/or encephalitis [5], [6]. One of the most commonly observed symptoms is an interstitial pneumonia [5], [6], [7], [8] that has led to additional names for OvLV including ovine progressive pneumonia virus and maedi-visna virus, from the Icelandic “maedi” for dyspnea. As with all known lentiviruses, there is currently no known way to eliminate OvLV infection, and no vaccine to completely prevent infection [9].

However, breed differences in susceptibility to OvLV suggest that it may be possible to breed animals with reduced susceptibility to the virus. It has been shown that reproducible breed differences exist in seroprevalence of OvLV across several studies done in different locations over many decades [10], [11], [12], [13], [14]. For example, Rambouillet sheep had lower odds of infection, Columbia sheep had higher odds of infection, and the recent composite breed Polypay were intermediate but with greater within-breed variation [10], [13], [15]. More recently, direct measurements of integrated provirus have confirmed breed differences not only in odds of infection but also relative immune control of viral levels once infected [15]. Specifically, Rambouillet sheep had lower proviral concentrations than Columbia sheep, and Polypay were again intermediate with greater within-breed variation [15]. Recently, mutations in one gene (*TMEM154: Transmembrane Protein 154*) were identified that contributed to relative susceptibility to OvLV in multiple breed backgrounds and widely differing environmental conditions [16]. However, the known mutations have not been shown to confer complete resistance to OvLV, and high prevalence has been observed in at least some flocks containing predominantly the less susceptible sheep *TMEM154* genotype [16]. Importantly, these and other genetic variants, once identified, can be used for effective marker-assisted selection even in the absence of pathogen exposure, which could greatly improve animal welfare and economic concerns when breeding for reduced susceptibility to OvLV.

Recent developments in genotyping technology should hasten identification of additional genes that could expand marker-assisted selection in sheep. Sufficiently dense marker systems (>50,000 single nucleotide polymorphisms (SNP)) have recently been released to allow examination of genome-wide variants related to phenotypes of interest [17]. These genome-wide association studies (GWAS) take advantage of the tendency of chromosome segments to be inherited together, which is referred to as linkage disequilibrium (LD). Specifically, when unobserved functional variants are inherited together with observed SNP, the observed SNP demonstrate association with the phenotype of interest. Further, it is widely acknowledged that association in multiple populations greatly enhances the value of genetic markers because it suggests that the markers are close enough to causal mutations to be in population-wide LD, a much higher standard than association in only one population [18]. Therefore, this study examined genome-wide association with both susceptibility to OvLV and control of OvLV replication in multiple breeds.

## Results

### Genotypes and Phenotypes

From the original set of 997 animals, sample quality control criteria eliminated 18 samples from additional analysis. Multi-dimensional scaling (MDS) identified 3 clusters corresponding to breed, and 15 animals were eliminated for outlier status (Figure S1). The remaining animals included Rambouillet (N = 399), Polypay (N = 423), and Columbia (N = 142). Pairwise population concordance (PPC) clusters matched breed for Columbia and Polypay but resulted in subdividing the Rambouillet breed into 2 clusters (Rambouillet_1 and Rambouillet_2). These clusters were used as stratification criteria for subsequent association analysis. Average genotyping call rate in the remaining animals was 98.06%. After initial screening for genotyping call rate by individual and by SNP, minor allele frequency, and Hardy-Weinberg equilibrium, there were slightly varying numbers of SNP for subsequent analysis depending on the animal set: 49,233 (all breeds), 50,275 (Rambouillet), 50,264 (Polypay), and 44,258 (Columbia).

Of the 964 animals that passed initial tests for genotype call quality, 95% had serological phenotypes and 96% had proviral concentration phenotypic data. The remaining blood samples were not of sufficient quality to report results. Among those individuals with serological results, 35% were positive for OvLV.

### Decline of Linkage Disequilibrium with Distance

To estimate the length of useful LD and thus provide genome-wide average expectation for the approximate distance from identified SNP within which underlying mutations might be found, LD was calculated for pairs of observed SNP. Decline of LD with physical distance in each breed is shown in Figure S2. The first distance bin exceeding the threshold of 1000 observations was 5–10 kilobase pairs (Kb), and this bin is reported in Figure S2 as 10 Kb. All individual breeds had r^2^<0.40 at 10 Kb and r^2^<0.25 at 35 Kb (Figure S2). The combined all-breeds set had lower LD of r^2^<0.35 at 10 Kb, r^2^<0.25 at 20 Kb, and r^2^<0.20 at 35 Kb (Figure S2).

### Genome-wide Association with Serological Status

Because OvLV infection is lifelong, concordance between serological status and direct viral measures of infection is high and serological status is a proxy for odds of infection in exposed populations [19]. Genome-wide association with serological status yielded 13 unique SNP, including 3 genome-wide significant and 10 genome-wide suggestive SNP (Table 1). For each SNP, results from the best-fitting mode-of-inheritance association model were reported in Table 1. Only one SNP (OAR1_185953850) was identified in separate analyses of both the all-breeds animal set and in an individual breed (Polypay). After accounting for *TMEM154* risk status as previously reported [16], the Table 1 SNP were associated with serologic status to varying degrees except OAR17_5388531, the SNP located within *TMEM154* (Table 2). Further, no additional SNP became genome-wide suggestive after accounting for *TMEM154* risk status.

**Table 1:** Genomic regions associated with susceptibility to OvLV.

SNP	Chr	Position (bp)	Animal Set	Best fitting model	NominalP-value	EmpiricalP-value	Odds Ratio	Genes within 100 Kb on either side
OAR1_185953850	1	172,600,491	All	additive	3.3×10^−8^	0.006	1.98	DPPA2**, DPPA4*
OAR1_185953850	1	172,600,491	Polypay	additive	6.5×10^−7^	0.048	2.60	DPPA2**, DPPA4*
OAR1_186779231	1	173,437,685	Polypay	dominant	1.7×10^−7^	0.012	4.45	−
OAR4_38205790	4	35,398,410	All	dominant	3.1×10^−6^	§	2.84	−
s54511	6	12,045,872	Polypay	additive	3.8×10^−6^	§	2.36	CAMK2D**
OAR7_82644472	7	75,607,567	Polypay	additive	5.7×10^−6^	§	2.53	GPHN**
OAR8_73555614	8	68,927,958	All	recessive	8.7×10^−6^	§	2.61	UTRN*, STX11
OAR8_88021348	8	82,158,519	Rambouillet	recessive	4.2×10^−6^	0.051	4.46	SYTL3**, GTF2H5**, DYNLT1*, TMEM181, EZR
OAR9_35880400	9	34,065,208	Polypay	additive	2.4×10^−6^	§	2.46	ST18
OAR17_5388531	17	4,862,358	All	additive	9.2×10^−7^	0.13	7.57	TMEM154**
s19031	17	12,016,817	All	additive	6.4×10^−6^	§	1.73	^−^
OAR18_9395406	18	9,449,325	All	genotypic	2.1×10^−6^	§	2.74	FUS*
OAR20_19572554	20	18,822,181	All	additive	2.0×10^−6^	§	1.80	SUPT3H**, RUNX2**
s56930	X	22,443,790	All	genotypic	7.2×10^−6^	§	2.43	^−^

§ : P>0.15

** : SNP located within gene

* : SNP located within 35 Kb of gene

**Table 2:** Genomic regions associated with OvLV serologic status comparing association with and without accounting for TMEM154 mutations.

SNP	Animal Set	NominalP-value (Without TMEM154)	NominalP-value (Accounting TMEM154)	EmpiricalP-value (Without TMEM154)	EmpiricalP-value (Accounting TMEM154)	Genes within 100 Kb on either side
OAR1_185953850	All	3.3×10^−8^	5.2×10^−6^	0.006	§	DPPA2**, DPPA4*
OAR1_185953850	Polypay	6.5×10^−7^	3.8×10^−5^	0.048	§	DPPA2**, DPPA4*
OAR1_186779231	Polypay	1.7×10^−7^	4.5×10^−6^	0.012	§	^−^
OAR4_38205790	All	3.1×10^−6^	1.0×10^−4^	§	§	^−^
s54511	Polypay	3.8×10^−6^	6.7×10^−4^	§	§	CAMK2D**
OAR7_82644472	Polypay	5.7×10^−6^	3.0×10^−5^	§	§	GPHN**
OAR8_73555614	All	8.7×10^−6^	5.8×10^−5^	§	§	UTRN*, STX11
OAR8_88021348	Rambouillet	4.2×10^−6^	3.2×10^−6^	0.051	0.052	SYTL3**, GTF2H5**, DYNLT1*, TMEM181, EZR
OAR9_35880400	Polypay	2.4×10^−6^	1.1×10^−5^	§	§	ST18
OAR17_5388531	All	9.2×10^−7^	§	0.13	§	TMEM154**
s19031	All	6.4×10^−6^	1.0×10^−4^	§	§	^−^
OAR18_9395406	All	2.1×10^−6^	1.1×10^−6^	§	§	FUS*
OAR20_19572554	All	2.0×10^−6^	3.4×10^−6^	§	§	SUPT3H**, RUNX2**
s56930	All	7.2×10^−6^	8.5×10^−5^	§	§	^−^

§ : P>0.15

** : SNP located within gene

* : SNP located within 35 Kb of gene

For each analysis, a second run was performed that dropped screening criteria including genotype call rate by individual and by SNP, minor allele frequency, and Hardy-Weinberg equilibrium. This technique had the potential to identify associated SNP that failed one or more of the screening criteria, for example common homozygous lethal genomic regions, but no additional genome-wide suggestive SNP were identified. No SNP reached genome-wide suggestive association in the Columbia analysis.

**Figure 1:**
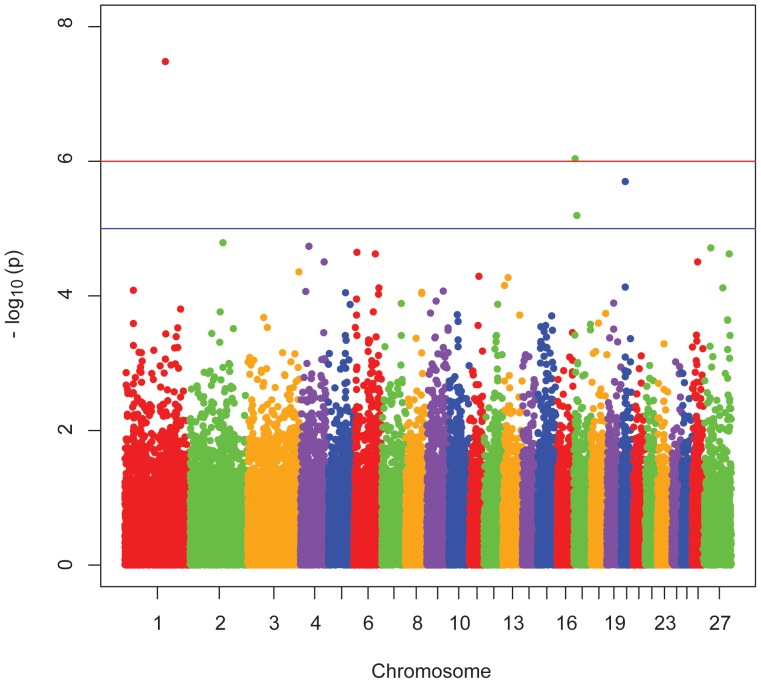
Manhattan plot for susceptibility to OvLV. The Manhattan plot shows nominal P-values from association with serological status by chromosomal position. Representative data from the all-breeds, additive mode of inheritance analysis are shown. The top red line shows a genome-wide significance threshold defined by nominal P-values of 1x10^-6^, which is P = 0.05/50,000. The lower blue line shows a genome-wide suggestive significance threshold defined by 1x10^-5^.

A Manhattan plot showing P-values arranged by chromosome position is shown in Figure 1. A series of Quantile-Quantile (Q-Q) plots showing observed versus expected P-value distributions are shown in Figures S3,S4,S5. Initial analysis showed population stratification (Figure S3), but most of this apparent population stratification was eliminated by accounting for SNP or genes identified in Table 1 (Figures S4,S5).

### Genome-wide Association with Proviral Concentration

The concentration of provirus in peripheral blood was used as a measure of control of infection, and it has been shown to be correlated with OvLV pathology [20]. Genome-wide association with proviral concentration identified 13 SNP, including 2 genome-wide significant and 11 genome-wide suggestive SNP (Table 3). For each SNP, results from the best-fitting mode-of-inheritance association model were reported in Table 3. The genotypic mean proviral concentrations, adjusted for age and breed, were reported in Table S1.

**Table 3:** Genomic regions associated with control of OvLV.

SNP	Chr	Position (bp)	Animal Set	Best fitting model	Nominal P-value	EmpiricalP-value	Genotypic Log_10_ Conc. Diff.	Genes within 100 Kb on either side
DU231007_156	3	58,955,947	Polypay	dominant	3.5×10^−6^	§	0.78	PAX8, IGK
OAR3_144283427	3	135,036,950	Polypay	genotypic	2.0×10^−6^	§	0.84	SLC11A2*
OAR3_144414855	3	135,043,018	Polypay	genotypic	2.0×10^−6^	§	0.84	SLC11A2**
s27054	5	6,250,424	Polypay	recessive	1.3×10^−6^	0.047	0.84	C19orf42**, TMEM38A*, NWD1, MED26, SLC35E1, CHERP
OAR9_10735564	9	10,702,461	Polypay	dominant	1.6×10^−6^	0.073	0.76	^−^
OAR9_10749779	9	10,725,321	Polypay	additive	1.5×10^−6^	0.069	0.92	^−^
s48118	9	14,589,931	Polypay	dominant	9.4×10^−6^	§	0.62	BAI1**, LOC529919*, ARC, RPL38
OAR13_56607666	13	52,062,577	All	additive	4.3×10^−6^	§	0.58	TGM6*
OAR18_5646940	18	5,926,257	Polypay	genotypic	2.5×10^−6^	§	0.68	MEF2A**
OAR18_5701234	18	5,984,107	Polypay	genotypic	2.6×10^−6^	§	0.68	MEF2A**
s65956	20	29,213,047	Rambouillet	dominant	5.9×10^−8^	0.001	0.89	ZNF192*, ZSCAN16*, ZNF165*, ZNF389*
OAR22_43742889	22	39,013,937	Polypay	additive	9.4×10^−6^	§	1.32	INPP5F**, MCMBP, BAG3
OAR23_40410527	23	38,376,040	All	additive	1.5×10^−6^	0.092	0.66	DLGAP1**

§ : P>0.15

** : SNP located within gene

* : SNP located within 35 Kb of gene

For each analysis, a second run was performed that dropped screening criteria including genotype call rate by individual and by SNP, minor allele frequency, and Hardy-Weinberg equilibrium. This technique had the potential to identify associated SNP that failed one or more of the screening criteria, but no additional genome-wide suggestive SNP were identified. The Columbia animal set included fewer animals with many fewer polymorphic SNP, and results from association with proviral concentration in Columbia sheep were reported in Table S2. Genotype frequencies of reported SNP were included in Table S3.

**Figure 2:**
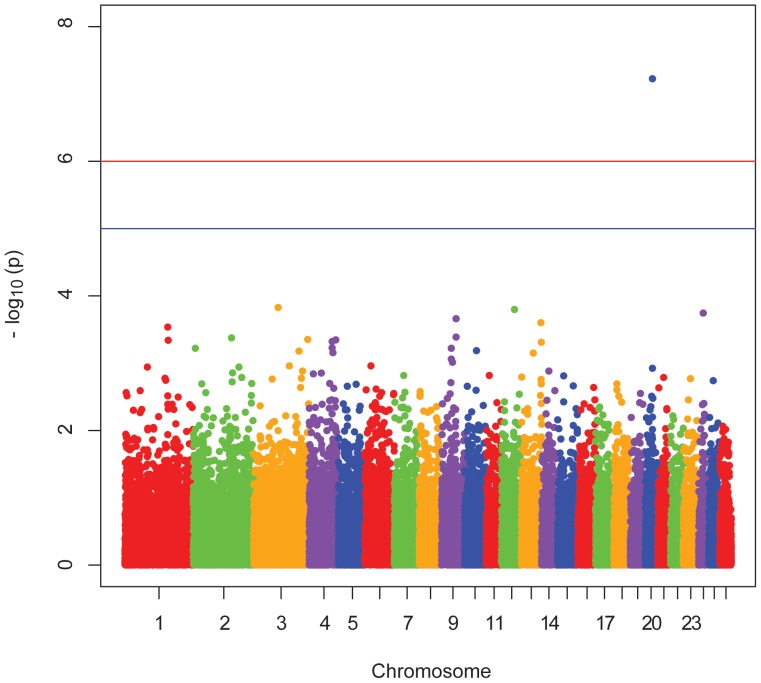
Manhattan plot for control of OvLV infection. The Manhattan plot shows nominal P-values from association with proviral concentration by chromosomal position. Representative data from the Rambouillet, dominant mode of inheritance analysis are shown. The top red line shows a genome-wide significance threshold defined by nominal P-values of 1x10^-6^, which is P = 0.05/50,000. The lower blue line shows a genome-wide suggestive significance threshold defined by nominal P-values of 1x10^-5^.

A Manhattan plot showing P-values arranged by chromosome position is shown in Figure 2. A series of Quantile-Quantile (Q-Q) plots showing observed versus expected P-value distributions are shown in Figures S6,S7. The initial analysis showed apparent population stratification (Figure S6), but most of this apparent population stratification was eliminated by accounting for SNP in Table 3 (Figure S7).

## Discussion

Breed differences in susceptibility to OvLV have long suggested genetic involvement, and a recent study identified *TMEM154* mutations as consistently associated with OvLV prevalence [16]. However, those mutations did not confer complete resistance, and some flocks with a high frequency of the less susceptible genotypes nonetheless have high OvLV prevalence. This GWAS identified multiple additional genomic regions as associated with OvLV susceptibility. Further, this was the first GWAS to examine control of OvLV replication to our knowledge, and multiple genomic regions were identified as associated with control of OvLV replication. The short LD length observed (Figure S2) was consistent with other reports in sheep [17] and implicated a smaller number of genes within each associated region than in many other mammal species [21], [22], [23], [24], [25]. For each region associated with either susceptibility or control of OvLV, the association suggested one or more underlying variants within the region had a functional relationship with some aspect of OvLV infection, replication, or transmission. Some regions contained promising candidate genes that suggested further biological hypotheses for specific gene product involvement. Below, we summarize major results as defined by empirical P-values and discuss future hypotheses of function to test for genes identified in connection with odds of infection and then for genes identified as associated with OvLV control of infection.

Genes involved in odds of infection could be used to select sheep with lower probabilities of natural infection, and the top GWAS markers provide important evidence for the involvement of several interesting genes. Only one gene had been identified previously as consistently associated with susceptibility to OvLV infection [16], and this GWAS also confirmed the association with *TMEM154* (empirical P = 0.13; Table 1). Further, the odds ratio was estimated at 7.57 (Table 1), which is consistent with prior data [16]. A recent study identified *TMEM154* in a human GWAS for asthma severity [26], suggesting that *TMEM154* may play a conserved role in airway immune responses. Only markers observed at empirical significance exceeding the *TMEM154* region harboring known mutations (empirical P<0.10) will be discussed further.

The locus most highly associated with odds of infection was a SNP in the *DPPA2* (*Developmental Pluripotency Associated 2*) gene which was associated in both the all-breeds analysis (empirical P = 0.006; Table 1) and in Polypays analyzed separately (empirical P = 0.048; Table 1). *DPPA2* and the closely related *DPPA4* (*Developmental Pluripotency Associated 4)* gene are expressed in embryo and germ line cells [27], and there are many potential mechanisms by which *DPPA2* or *DPPA4* may be involved in OvLV infection. Both *DPPA2* and the nearby *DPPA4* play essential roles in lung development and formation [28], [29], suggesting differential lung development in sheep may play a role in OvLV susceptibility. However, OvLV has been found in semen and in the female reproductive tract of sheep [30], [31], and the possibility of sexual transmission and/or paternal transmission of the virus are important unanswered questions. If OvLV is sexually or paternally transmitted, germline expressed *DPPA2* may play a role in some aspect of transmission. A third possibility is differential development of the immune system. Since *DPPA2* is expressed during embryonic development, it is possible that *DPPA2* variants may influence the development or maintenance of the immune system. The SheepQTLdb [32], [33] shows a QTL for *Haemonchus contortus* fecal egg count [34] that overlaps this region. An intriguing possibility would involve *DPPA2* influencing both OvLV and parasitic infection through alterations in immune system development, since alterations in embryonic and/or white blood cell development could influence formation and/or maintenance of immune responses.

A second locus associated with OvLV susceptibility after correction for multiple testing was identified in Polypays (OAR1_186779231; empirical P = 0.012) and was located 837 Kb distal on ovine chromosome 1 from the SNP in *DPPA2* (Table 1). This distance is considerably further than the genome-wide average decline of LD would suggest (Figure S2), but there is nonetheless appreciable LD between the markers (Table S4). In Polypays, these two SNP had r^2^ = 0.40, and in all breeds examined these SNP had r^2^ = 0.23 (Table S4). It is possible that the associations of both SNP reflect a single underlying gene through long-range LD due to admixture in the composite breed formation of the Polypay population [35], [36]. Alternatively, a separate associated genetic factor could be present, though the current version of the sheep genome contains no annotated genes near OAR1_186779231. Nonetheless, the example of the callipyge muscular hypertrophy in sheep demonstrates that even a single nucleotide change in a gene desert can have a dramatic impact [37], [38].

A separate region associated with odds of infection included *SYTL3* (*Synaptotagmin-Like 3)* on ovine chromosome 8, and this region had the second largest odds ratio after *TMEM154* (Table 1). *SYTL3* is a peripheral membrane protein that interacts with *RAB27A* (*RAB27A, member RAS oncogene family*) and is thought to play a role in vesicular trafficking [39], [40]. To our knowledge there has been no report of *SYTL3* associated with any lentivirus infection in any mammal, but *RAB27A* is required for enveloped virus assembly of human cytomegalovirus [41]. *SYTL3* may regulate *RAB27A* and thereby interfere with viral assembly. Further, *RAB27A* is a known negative regulator of phagocytosis [42], and is involved in exosome synthesis [43]. As such, *SYTL3* could also influence either of those processes. While the current version of the sheep genome also shows SNP OAR8_88021348 within transcriptional regulator *GTF2H5* (*General Transcription Factor IIH, polypeptide 5*), the gene order does not agree with other mammal genomes including cow, human, dog, and mouse, and it may well be a mis-assembly in the current version of the sheep genome.

A further analysis of genomic regions associated with serological status incorporated data on risk status defined by known *TMEM154* diplotypes [16]. In this analysis, SNP near *DPPA2*/*DPPA4* dropped from genome-wide significant to genome-wide suggestive, but the SNP in *SYTL3* retained its level of significance (Table 2). This strengthened the case for association between serological status and an underlying variant near *SYTL3*. It is possible that part of the association between serological status and SNP in or near *DPPA2*/*DPPA4* was due to random association between genotypes at *TMEM154* and *DPPA2*/*DPPA4*. However, this type of analysis tested association of SNP in or near the *DPPA2*/*DPPA4* region averaged over all genotypes of *TMEM154*. Thus, it could underrepresent association in the presence of epistasis, where differential association exists at one locus by genotype at another. However, the relatively small overlap between genotype frequencies of SNP near *DPPA2*/*DPPA4* and risk diplotypes of*TMEM154* suggested a larger sample including additional animals would be required to perform a thorough test for genetic interaction.

As well as adding more genomic regions associated with odds of infection, this was the first genome-wide study to examine association with the control of viral replication as measured by proviral concentration, to our knowledge. The most significant SNP (s65956; empirical P = 0.001) was located in a cluster of zinc finger genes on ovine chromosome 20 (Table 3). Zinc finger proteins are known to have undergone duplication and divergent positive selection in the DNA-binding zinc finger domain following challenge by new retroviruses during mammalian evolution [44], [45]. This suggests the hypothesis that certain zinc finger proteins might interfere with lentiviral replication, and indeed that has recently been shown with the *Zinc Finger Antiviral Protein* (*ZAP*) and HIV-1 [46]. However, to our knowledge none of the zinc finger proteins in the cluster identified here has ever been shown to be associated with any mammalian lentiviral infection.

Another empirically significant SNP (s27054; P = 0.047) was located within both *C19orf42* ([human] *Chromosome 19 Open Reading Frame 19*) and *TMEM38A* (*Transmembrane Protein 38A*) (Table 3). Not much is known about either *C19orf42* or *TMEM38A* at this time. *C19orf42* is a short (75 amino acid) open reading frame with similarity to Yos1, a yeast protein required for transport between endoplasmic reticulum and Golgi complex [47]. As such, *C19orf42* could be involved in efficiency of viral packaging, but data to demonstrate such involvement are currently lacking. *TMEM38A* encodes a protein associated with vascular smooth muscle control of blood pressure [48]. It is possible that *TMEM38A* could be involved by an as-yet undetermined mechanism. Much work remains to be done to elucidate the potential roles of these genes in limiting OvLV replication.

Finally, there were two additional regions with suggestive empirical association (P<0.10) with proviral concentration. The first included two SNP on ovine chromosome 9 in Polypays (Table 3), but no genes are annotated in that region in the current pre-release version of the sheep genome. The other is SNP OAR23_40410527 located within the *DLGAP1* (*Discs Large (Drosophila) Homolog-Associated Protein 1*) gene (Table 3). The product of DLGAP1 is a primary interacting protein of *DLG1* and is believed to function by holding *DLG1* in place at cell junctions [49], [50], [51]. *DLG1* is a strong negative regulator of HIV-1 infectivity, such that depletion of *DLG1* enhances HIV infectivity 5–6 fold [52]. The allelic variant(s) of *DLGAP1* in sheep may physically interfere with *DLG1* positioning and/or function. Though *DLGAP1* was associated with mouse survival following influenza H5N1 infection [53], possibly mediated through its interaction with *DLG1* [54], to our knowledge *DLGAP1* has not been reported to have a role in lentiviral infection in any mammal to date.

In conclusion, this GWAS found many additional genomic regions besides *TMEM154* associated with odds of infection, and this was the first genome-wide study to provide regions associated with OvLV control as measured by proviral concentration. These results confirmed that *TMEM154* is an important component of host susceptibility to OvLV, but there were many additional factors that contributed to genetic differences in susceptibility to and control of OvLV infection. These included many genes never previously associated with lentiviral infection, which may extend structural and/or regulatory networks implicated in lentiviral transmission, infection, and/or replication. These genes provide targets for additional investigation into lentiviral infection that may generalize beyond OvLV to other members of the lentiviral family. Further, the associated genes provide the basis for additional work to identify genetic markers associated with odds of OvLV infection and proviral concentration. As has already been done with *TMEM154*, such markers may be used to select sheep with lower odds and/or improved control of OvLV infection. Finally, they may also be of great interest for further study in goats, which are host to the closely related caprine encephalitis arthritis virus.

## Materials and Methods

### Ethics Statement

All animal care and handling procedures were reviewed and approved by the Washington State University Institutional Animal Care and Use Committee (Permit Number: 3171) and/or by the U.S. Sheep Experiment Station Animal Care and Use Committee (Permit Numbers: 10-06, 10-07). All efforts were made to minimize any discomfort during blood sampling.

### Populations and Phenotypes

Flocks were chosen for study with high historical OvLV seroprevalence among mature ewes [15]. Specifically, whole blood was collected from ewes of Rambouillet (N = 414), Polypay (N = 438), and Columbia (N = 145) breeds, ages 1–5 years, from the U.S. Sheep Experiment Station. These animals were managed similarly but bred separately in pure breed groups. Blood was processed for serum and peripheral blood leukocytes as previously described [19]. A subset of 365 older ewes (ages 3–5) had been used as validation animals in a previous study on *TMEM154* [16]. Serologic data were collected using a competitive ELISA assay (VMRD Inc., Pullman, WA) to detect anti-OvLV antibodies in sheep [55]. DNA was extracted from peripheral blood leukocytes and OvLV proviral concentrations were determined by a validated qPCR method [19].

### Genotyping

Blood was collected by jugular venipuncture into EDTA-coated vacutainer tubes. DNA was isolated using the Invitrogen GeneCatcher™ gDNA 3–10 ml Blood Kit as per manufacturers' instructions (Life Technologies, Carlsbad, CA). The DNAs were checked for quality and quantity using an ND-1000 spectrophotometer (Nanodrop, Wilmington, DE) and equilibrated to 50 ng/µl for genotyping. Genotyping services were provided by Geneseek Inc. (Lincoln, NE) using the OvineSNP50 Infinium BeadChip (Illumina Inc., San Diego, CA) with a set of 54,977 SNP designed by the International Sheep Genome Consortium [17].

### Statistical analysis

#### Genotypic quality control and clustering

Initial quality control criteria included low genotype call rates and high genotype identity to any other sample. Samples with low genotype call rates (<97%) were removed from additional analysis. PLINK identity-by-state analysis identified samples that could have been involved in label errors (as determined by IBS distances >0.95) for removal from additional analysis. Multidimensional scaling (MDS) was performed in the PLINK software package (http://pngu.mgh.harvard.edu/purcell/plink/) written by Shaun Purcell [56] to check for genotypic outliers. MDS was performed on a reduced set of markers in approximate linkage equilibrium from animals of each breed separately. Outlier animals were removed to reduce population stratification. Then, a pairwise population concordance test (–ppc option) of the hypothesis that each animal was from a separate breeding population was performed using a P-value of 10^−7^, which is P = 0.05 after Bonferroni-correction for the total number of pairwise comparisons between animals. Where the resulting clusters contained additional information compared to recorded breed data, the clusters were included in addition to breed as stratification categories for later association analyses.

#### Linkage Disequilibrium Versus Genomic Distance

Analysis of high density SNP data was performed using PLINK v1.06 [56]. A preliminary screen was performed to eliminate SNP with minor allele frequencies less than 0.10 from comparisons used to calculate average LD. LD within each breed was calculated with settings to include comparisons of all SNP within 5 Mbp of each other and minimum reported r^2^ adjusted to 0.0 to include even low LD comparisons. These r^2^ values were analyzed by distance bins with SAS 9.2 (SAS Institute, Cary, NC). Distance bins were calculated for each 5 Kbp interval beginning with 0–5 Kbp. Mean r^2^ was only included in analysis if a bin contained at least 1000 values to assure each reported mean was well-estimated.

#### Association analysis

The OvLV positive or negative status as measured by cELISA was analyzed using separate logistic models for the minor allele of each SNP in PLINK to account for breed and pairwise population concordance clusters, for animal age as a covariate, and for the SNP minor allele. Log_10_-transformed proviral concentrations were analyzed using similar separate general linear models for the minor allele of each SNP in PLINK to account for breed and pairwise population concordance clusters, for animal age as a covariate, and for the SNP minor allele. For both logistic and linear analyses, PLINK screening criteria were employed including missingness by individual (0.1), missingness by marker (0.03), minor allele frequency (0.01), and Hardy-Weinberg equilibrium (0.000001, which is P = 0.05 Bonferroni-corrected for 50,000 SNP tests). Genome-wide significance was defined by empirical P≤0.05. Genome-wide suggestive results were defined by nominal P-values < 1×10^−5^ [57]. Both logistic and linear analyses were performed using association models including additive allelic, genotypic 2 degree-of-freedom, dominant, and recessive. Family structure was addressed for both logistic and general linear models using permutation within sire families of 10 or more genotyped offspring; the remaining sire families with fewer than 10 offspring were grouped together for permutation purposes. One thousand permutations were used to obtain each empirical P-value. Additional analyses of serologic data included the presence or absence of risk haplotypes as defined by Heaton et al. [16] as covariates in the respective association models. Visualization of association data in manhattan and quantile-quantile plots was performed using a script generously provided by Dr. Stephen Turner (http://gettinggeneticsdone.blogspot.com/2011/04/annotated-manhattan-plots-and-qq-plots.html, viewed on 11-15-11) using the R environment [58]. Since PLINK only reports regression coefficients as a measure of effect size for linear regression, SAS 9.2 (SAS Institute, Cary, NC) was used to run similar genotypic models in the general linear models procedure to obtain largest adjusted genotypic mean differences in log_10_−transformed proviral concentration as a measure of effect size for control of OvLV.

## Supporting Information

Figure S1 **Multidimensional scaling of genotypes showing 3 breed clusters of animals included in analysis.** Columbias are included in the top cluster, Polypays in the bottom right cluster, and Rambouillets in the bottom left cluster. The clustering of individuals by breed is clear even from these related breeds. The Columbia breed was developed pre-1920 with ½ Rambouillet composition [59]. The Polypay breed was developed in the 1970s with ¼ Rambouillet composition [36].
(PDF)

Figure S2 **Decline of linkage disequilibrium with distance by breed.**
(PDF)

Figure S3 **Quantile-Quantile plot for odds of infection.** Quantile-quantile plots from association with serological status, where the red line shows the expected distribution. Representative data from the all-breeds, additive mode of inheritance analysis are shown. The results show deviation from the expected distribution indicating population stratification by factors unaccounted in the analytic model, which could include frequencies of underlying mutations for susceptibility loci that differ between seropositive and seronegative individuals.
(PDF)

Figure S4 **Quantile-Quantile plot for odds of infection conditioned on TMEM154 risk status.** A second analysis conditioned on TMEM154 risk status shows an observed distribution closer to expected than the primary analysis, but still does not account for the majority of apparent population stratification.
(PDF)

Figure S5 **Quantile-Quantile plot for odds of infection conditioned on** **Table 1** **SNP.** A third analysis conditioned on all the SNP in Table 1 shows a distribution much closer to expected, demonstrating that host genetic factors tracked by these SNP account for the majority of apparent population stratification.
(PDF)

Figure S6 **Quantile-Quantile plot for control of viral replication.** Quantile-quantile plot from association with proviral concentration, where the red line shows the expected distribution. Representative data from the Rambouillet, dominant mode of inheritance analysis are shown. The results show deviation from the expected distribution indicating population stratification by factors unaccounted in the analytic model, which could include frequencies of underlying mutations for susceptibility loci that differ by proviral concentration.
(PDF)

Figure S7 **Quantile-Quantile plot for control of viral replication conditioned on** **Table 3** **SNP.** A second analysis was performed by conditioning on all the SNP in Table 3, minus close equivalents on the same chromosome (r^2^>0.8; removed to prevent inestimable multicollinearity) for which only the best P-value SNP was retained from each pair. This analysis shows a distribution much closer to expected, demonstrating that host genetic factors tracked by these SNP account for the majority of apparent population stratification.
(PDF)

Table S1 **Adjusted genotypic mean proviral concentrations for** **Table 3** **SNP.** Mean proviral concentrations by genotype, adjusted for age and breed.
(DOC)

Table S2 **Genomic regions from Columbia breed associated with proviral concentration of ovine lentivirus.** Genomic regions from Columbia breed associated with proviral concentration of ovine lentivirus.
(DOC)

Table S3 **Genotype frequencies by animal set for SNP from** **Tables 1** **,** **2** **, and S2.**
(XLSX)

Table S4 **Linkage disequilibrium by animal set between SNP from** **Tables 1** **,** **2** **, and S2 by chromosome.**
(XLSX)

## References

[1] Leroux C, Cruz JC, Mornex JF (2010) SRLVs: a genetic continuum of lentiviral species in sheep and goats with cumulative evidence of cross species transmission. Curr HIV Res 8: 94–100. 20210785 10.2174/157016210790416415

[2] Blacklaws B, Harkiss GD (2010) Small ruminant lentiviruses and human immunodeficiency virus: cousins that take a long view. Curr HIV Res 8: 26–52. 20210779 10.2174/157016210790416406

[3] Cutlip RC, Lehmkuhl HD, Sacks JM, Weaver AL (1992) Seroprevalence of ovine progressive pneumonia virus in sheep in the United States as assessed by analyses of voluntarily submitted samples. Am J Vet Res 53: 976–979. 1320816

[4] InfoSheet (2003) Ovine Progressive Pneumonia: Awareness, Management, and Seroprevalence. USDA-APHIS-Veterinary Services. 1–4.

[5] Narayan O, Kennedy-Stoskopf S, Zink MC (1988) Lentivirus-host interactions: lessons from visna and caprine arthritis-encephalitis viruses. Ann Neurol 23 Suppl S95–100. 2831808 10.1002/ana.410230725

[6] De Boer GF, Terpstra C, Houwers DJ, Hendriks J (1979) Studies in epidemiology of maedi/visna in sheep. Res Vet Sci 26: 202–208. 233619

[7] Torsteinsdottir S, Andresdottir V, Arnarson H, Petursson G (2007) Immune response to maedi-visna virus. Front Biosci 12: 1532–1543. 17127400 10.2741/2166

[8] Blacklaws BA (2012) Small ruminant lentiviruses: immunopathogenesis of visna-maedi and caprine arthritis and encephalitis virus. Comp Immunol Microbiol Infect Dis 35: 259–269. 22237012 10.1016/j.cimid.2011.12.003

[9] Reina R, Berriatua E, Lujan L, Juste R, Sanchez A, et al (2009) Prevention strategies against small ruminant lentiviruses: an update. Vet J 182: 31–37. 18755622 10.1016/j.tvjl.2008.05.008

[10] Gates NL, Winward LD, Gorham JR, Shen DT (1978) Serologic survey of prevalence of ovine progressive pneumonia in Idaho range sheep. J Am Vet Med Assoc 173: 1575–1577. 218917

[11] Cutlip RC, Lehmkuhl HD, Brogden KA, Sacks JM (1986) Breed susceptibility to ovine progressive pneumonia (maedi/visna) virus. Vet Microbiol 12: 283–288. 3776096 10.1016/0378-1135(86)90057-x

[12] Houwers DJ, Visscher AH, Defize PR (1989) Importance of ewe/lamb relationship and breed in the epidemiology of maedi-visna virus infections. Res Vet Sci 46: 5–8. 2537992

[13] Snowder GD, Gates NL, Glimp HA, Gorham JR (1990) Prevalence and effect of subclinical ovine progressive pneumonia virus infection on ewe wool and lamb production. J Am Vet Med Assoc 197: 475–479. 2170310

[14] Keen JE, Hungerford LL, Wittum TE, Kwang J, Littledike ET (1997) Risk factors for seroprevalence of ovine lentivirus in breeding ewe flocks in Nebraska, USA. Prev Vet Med 30: 81–94. 9234413 10.1016/s0167-5877(96)01121-x

[15] Herrmann-Hoesing LM, White SN, Mousel MR, Lewis GS, Knowles DP (2008) Ovine progressive pneumonia provirus levels associate with breed and Ovar-DRB1. Immunogenetics 60: 749–758. 18797863 10.1007/s00251-008-0328-9

[16] Heaton MP, Clawson ML, Chitko-McKown CG, Leymaster KA, Smith TP, et al (2012) Reduced Lentivirus Susceptibility in Sheep with TMEM154 Mutations. PLoS Genet 8: e1002467. 22291605 10.1371/journal.pgen.1002467 PMC3266874

[17] Kijas JW, Lenstra JA, Hayes B, Boitard S, Porto Neto LR, et al (2012) Genome-wide analysis of the world's sheep breeds reveals high levels of historic mixture and strong recent selection. PLoS Biol 10: e1001258. 22346734 10.1371/journal.pbio.1001258 PMC3274507

[18] Slatkin M (2008) Linkage disequilibrium–understanding the evolutionary past and mapping the medical future. Nat Rev Genet 9: 477–485. 18427557 10.1038/nrg2361 PMC5124487

[19] Herrmann-Hoesing LM, White SN, Lewis GS, Mousel MR, Knowles DP (2007) Development and validation of an ovine progressive pneumonia virus quantitative PCR. Clin Vaccine Immunol 14: 1274–1278. 17699832 10.1128/CVI.00095-07 PMC2168119

[20] Herrmann-Hoesing LM, Noh SM, White SN, Snekvik KR, Truscott T, et al (2009) Peripheral ovine progressive pneumonia provirus levels correlate with and predict histological tissue lesion severity in naturally infected sheep. Clin Vaccine Immunol 16: 551–557. 19261772 10.1128/CVI.00459-08 PMC2668279

[21] Kim ES, Kirkpatrick BW (2009) Linkage disequilibrium in the North American Holstein population. Anim Genet 40: 279–288. 19220233 10.1111/j.1365-2052.2008.01831.x

[22] Qanbari S, Pimentel EC, Tetens J, Thaller G, Lichtner P, et al (2010) The pattern of linkage disequilibrium in German Holstein cattle. Anim Genet 41: 346–356. 20055813 10.1111/j.1365-2052.2009.02011.x

[23] Badke YM, Bates RO, Ernst CW, Schwab C, Steibel JP (2012) Estimation of linkage disequilibrium in four US pig breeds. BMC Genomics 13: 24. 22252454 10.1186/1471-2164-13-24 PMC3269977

[24] Sutter NB, Eberle MA, Parker HG, Pullar BJ, Kirkness EF, et al (2004) Extensive and breed-specific linkage disequilibrium in Canis familiaris. Genome Res 14: 2388–2396. 15545498 10.1101/gr.3147604 PMC534662

[25] Consortium TIH (2005) A haplotype map of the human genome. Nature 437: 1299–1320. 16255080 10.1038/nature04226 PMC1880871

[26] Slager RE, Li X, Meyers DA, Bleecker ER (2011) Recent developments in the genetics of asthma susceptibility and severity. In: Chung KF, Bel E H, Wenzel SE, editors. Difficult-to-Treat Severe Asthma. Plymouth, UK: European Respiratory Society. 82–96.

[27] Du J, Lin G, Nie ZY, Lu GX (2004) [Molecular cloning and characterization analysis of HPESCRG1, a novel gene expressed specifically in human embryonic stem cell]. Zhonghua Yi Xue Yi Chuan Xue Za Zhi 21: 542–547. 15583978

[28] Madan B, Madan V, Weber O, Tropel P, Blum C, et al (2009) The pluripotency-associated gene Dppa4 is dispensable for embryonic stem cell identity and germ cell development but essential for embryogenesis. Mol Cell Biol 29: 3186–3203. 19332562 10.1128/MCB.01970-08 PMC2682008

[29] Nakamura T, Nakagawa M, Ichisaka T, Shiota A, Yamanaka S (2011) Essential roles of ECAT15-2/Dppa2 in functional lung development. Mol Cell Biol 31: 4366–4378. 21896782 10.1128/MCB.05701-11 PMC3209334

[30] Cortez-Romero C, Fieni F, Russo P, Pepin M, Roux C, et al (2011) Presence of Maedi Visna virus (MVV)-proviral DNA in the genital tissues of naturally infected ewes. Reprod Domest Anim 46: e1–6. 20403133 10.1111/j.1439-0531.2010.01608.x

[31] Peterson K, Brinkhof J, Houwers DJ, Colenbrander B, Gadella BM (2008) Presence of pro-lentiviral DNA in male sexual organs and ejaculates of small ruminants. Theriogenology 69: 433–442. 18037482 10.1016/j.theriogenology.2007.10.013

[32] Hu ZL, Fritz ER, Reecy JM (2007) AnimalQTLdb: a livestock QTL database tool set for positional QTL information mining and beyond. Nucleic Acids Res 35: D604–609. 17135205 10.1093/nar/gkl946 PMC1781224

[33] Hu ZL, Reecy JM (2007) Animal QTLdb: beyond a repository. A public platform for QTL comparisons and integration with diverse types of structural genomic information. Mamm Genome 18: 1–4. 17245610 10.1007/s00335-006-0105-8

[34] Marshall K, Maddox JF, Lee SH, Zhang Y, Kahn L, et al (2009) Genetic mapping of quantitative trait loci for resistance to Haemonchus contortus in sheep. Anim Genet 40: 262–272. 19291139 10.1111/j.1365-2052.2008.01836.x

[35] Seldin MF (2007) Admixture mapping as a tool in gene discovery. Curr Opin Genet Dev 17: 177–181. 17466511 10.1016/j.gde.2007.03.002 PMC3146309

[36] Hulet CV, Ercanbrack SK, Knight AD (1984) Development of the Polypay breed of sheep. J Anim Sci 58: 15–24. 6698896 10.2527/jas1984.58115x

[37] Cockett NE, Jackson SP, Shay TL, Farnir F, Berghmans S, et al (1996) Polar overdominance at the ovine callipyge locus. Science 273: 236–238. 8662506 10.1126/science.273.5272.236

[38] Freking BA, Murphy SK, Wylie AA, Rhodes SJ, Keele JW, et al (2002) Identification of the single base change causing the callipyge muscle hypertrophy phenotype, the only known example of polar overdominance in mammals. Genome Res 12: 1496–1506. 12368241 10.1101/gr.571002 PMC187527

[39] Kuroda TS, Fukuda M, Ariga H, Mikoshiba K (2002) The Slp homology domain of synaptotagmin-like proteins 1–4 and Slac2 functions as a novel Rab27A binding domain. J Biol Chem 277: 9212–9218. 11773082 10.1074/jbc.M112414200

[40] Fukuda M, Mikoshiba K (2001) Synaptotagmin-like protein 1–3: a novel family of C-terminal-type tandem C2 proteins. Biochem Biophys Res Commun 281: 1226–1233. 11243866 10.1006/bbrc.2001.4512

[41] Fraile-Ramos A, Cepeda V, Elstak E, van der Sluijs P (2010) Rab27a is required for human cytomegalovirus assembly. PloS One 5: e15318. 21170347 10.1371/journal.pone.0015318 PMC2999566

[42] Yokoyama K, Kaji H, He J, Tanaka C, Hazama R, et al (2011) Rab27a negatively regulates phagocytosis by prolongation of the actin-coating stage around phagosomes. J Biol Chem 286: 5375–5382. 21169636 10.1074/jbc.M110.171702 PMC3037650

[43] Ostrowski M, Carmo NB, Krumeich S, Fanget I, Raposo G, et al. (2010) Rab27a and Rab27b control different steps of the exosome secretion pathway. Nat Cell Biol 12: : 19–30; sup 11–13. 10.1038/ncb2000 19966785

[44] Emerson RO, Thomas JH (2009) Adaptive evolution in zinc finger transcription factors. PLoS Genet 5: e1000325. 19119423 10.1371/journal.pgen.1000325 PMC2604467

[45] Thomas JH, Schneider S (2011) Coevolution of retroelements and tandem zinc finger genes. Genome Res 21: 1800–1812. 21784874 10.1101/gr.121749.111 PMC3205565

[46] Zhu Y, Chen G, Lv F, Wang X, Ji X, et al (2011) Zinc-finger antiviral protein inhibits HIV-1 infection by selectively targeting multiply spliced viral mRNAs for degradation. Proc Natl Acad Sci U S A 108: 15834–15839. 21876179 10.1073/pnas.1101676108 PMC3179061

[47] Heidtman M, Chen CZ, Collins RN, Barlowe C (2005) Yos1p is a novel subunit of the Yip1p-Yif1p complex and is required for transport between the endoplasmic reticulum and the Golgi complex. Mol Biol Cell 16: 1673–1683. 15659647 10.1091/mbc.E04-10-0873 PMC1073651

[48] Yamazaki D, Tabara Y, Kita S, Hanada H, Komazaki S, et al (2011) TRIC-A channels in vascular smooth muscle contribute to blood pressure maintenance. Cell Metab 14: 231–241. 21803293 10.1016/j.cmet.2011.05.011

[49] Satoh K, Yanai H, Senda T, Kohu K, Nakamura T, et al (1997) DAP-1, a novel protein that interacts with the guanylate kinase-like domains of hDLG and PSD-95. Genes Cells 2: 415–424. 9286858 10.1046/j.1365-2443.1997.1310329.x

[50] Kim E, Naisbitt S, Hsueh YP, Rao A, Rothschild A, et al (1997) GKAP, a novel synaptic protein that interacts with the guanylate kinase-like domain of the PSD-95/SAP90 family of channel clustering molecules. J Cell Biol 136: 669–678. 9024696 10.1083/jcb.136.3.669 PMC2134290

[51] Takeuchi M, Hata Y, Hirao K, Toyoda A, Irie M, et al (1997) SAPAPs. A family of PSD-95/SAP90-associated proteins localized at postsynaptic density. J Biol Chem 272: 11943–11951. 9115257 10.1074/jbc.272.18.11943

[52] Perugi F, Muriaux D, Ramirez BC, Chabani S, Decroly E, et al (2009) Human Discs Large is a new negative regulator of human immunodeficiency virus-1 infectivity. Mol Biol Cell 20: 498–508. 18946087 10.1091/mbc.E08-02-0189 PMC2613124

[53] Boon AC, deBeauchamp J, Hollmann A, Luke J, Kotb M, et al (2009) Host genetic variation affects resistance to infection with a highly pathogenic H5N1 influenza A virus in mice. J Virol 83: 10417–10426. 19706712 10.1128/JVI.00514-09 PMC2753106

[54] Javier RT, Rice AP (2011) Emerging Theme: Cellular PDZ Proteins as Common Targets of Pathogenic Viruses. J Virol 85: 11544–11556. 21775458 10.1128/JVI.05410-11 PMC3209276

[55] Herrmann LM, Cheevers WP, Marshall KL, McGuire TC, Hutton MM, et al (2003) Detection of serum antibodies to ovine progressive pneumonia virus in sheep by using a caprine arthritis-encephalitis virus competitive-inhibition enzyme-linked immunosorbent assay. Clin Diagn Lab Immunol 10: 862–865. 12965917 10.1128/CDLI.10.5.862-865.2003 PMC193903

[56] Purcell S, Neale B, Todd-Brown K, Thomas L, Ferreira MA, et al (2007) PLINK: a tool set for whole-genome association and population-based linkage analyses. Am J Hum Genet 81: 559–575. 17701901 10.1086/519795 PMC1950838

[57] Hindorff LA, Sethupathy P, Junkins HA, Ramos EM, Mehta JP, et al (2009) Potential etiologic and functional implications of genome-wide association loci for human diseases and traits. Proc Natl Acad Sci U S A 106: 9362–9367. 19474294 10.1073/pnas.0903103106 PMC2687147

[58] Team RDC (2011) R: A Language and Environment for Statistical Computing. Vienna, Austria: R Foundation for Statistical Computing.

[59] Terrill CE (1958) Fifty Years of Progress in Sheep Breeding. J Anim Sci 17: 944–959.

