# Supplementary figures and images for: Genome-Wide Association Identifies Multiple Genomic Regions Associated with Susceptibility to and Control of Ovine Lentivirus
Source: PLoS One. 2012 Oct 17;7(10):e47829. doi: 10.1371/journal.pone.0047829 (PMC3474742; doi:10.1371/journal.pone.0047829)

**Figure S2: Multidimensional Scaling after Screening Outliers**

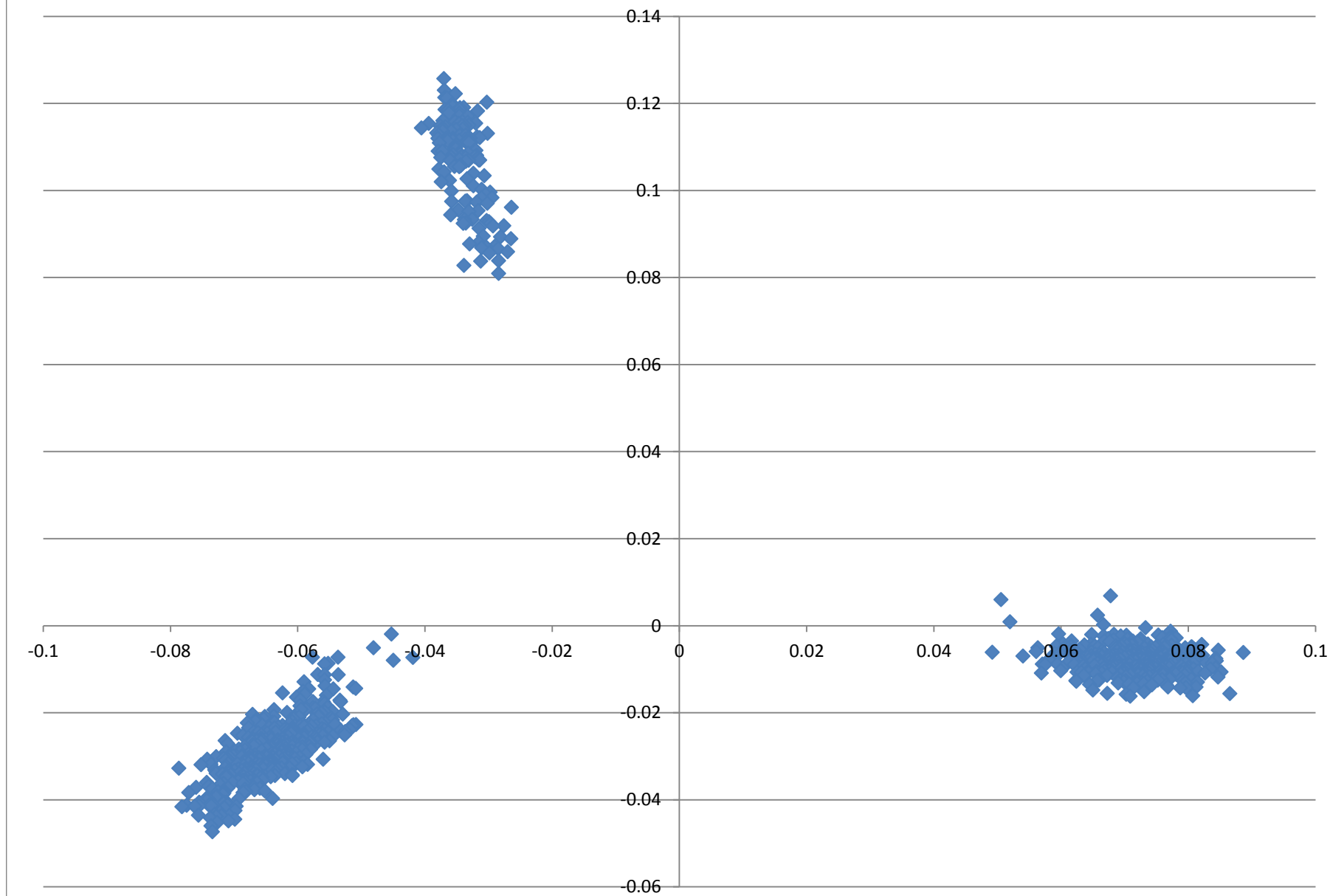

Supplement: Figure S1 — Multidimensional scaling of genotypes showing 3 breed clusters of animals included in analysis. Columbias are included in the top cluster, Polypays in the bottom right cluster, and Rambouillets in the bottom left cluster. The clustering of individuals by breed is clear even from these related breeds. The Columbia breed was developed pre-1920 with ½ Rambouillet composition [59]. The Polypay breed was developed in the 1970s with ¼ Rambouillet composition [36]. (PDF) [file pone.0047829.s001.pdf]

**Figure S3 - Decline of LD with distance by breed set**

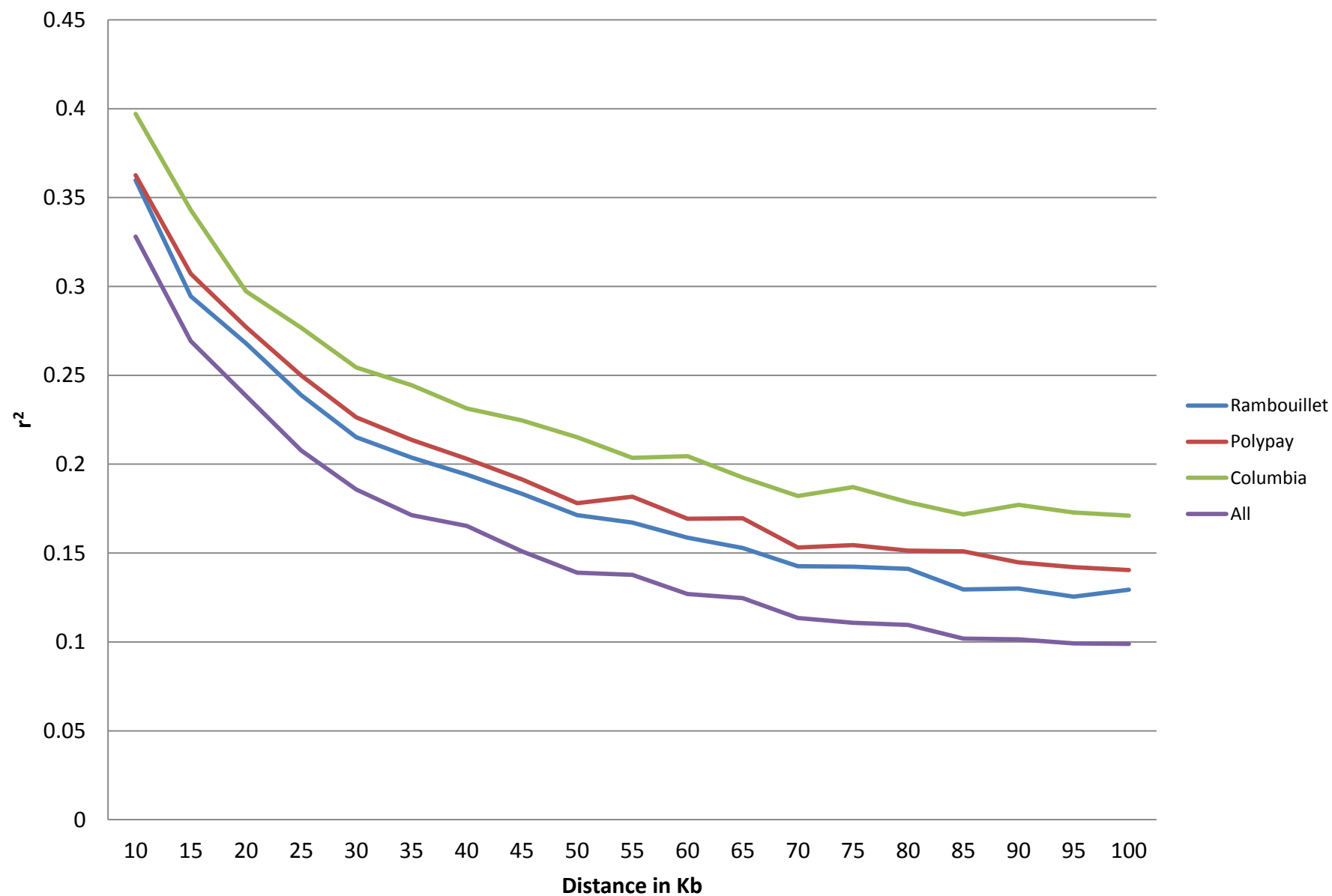

Supplement: Figure S2 — Decline of linkage disequilibrium with distance by breed. (PDF) [file pone.0047829.s002.pdf]

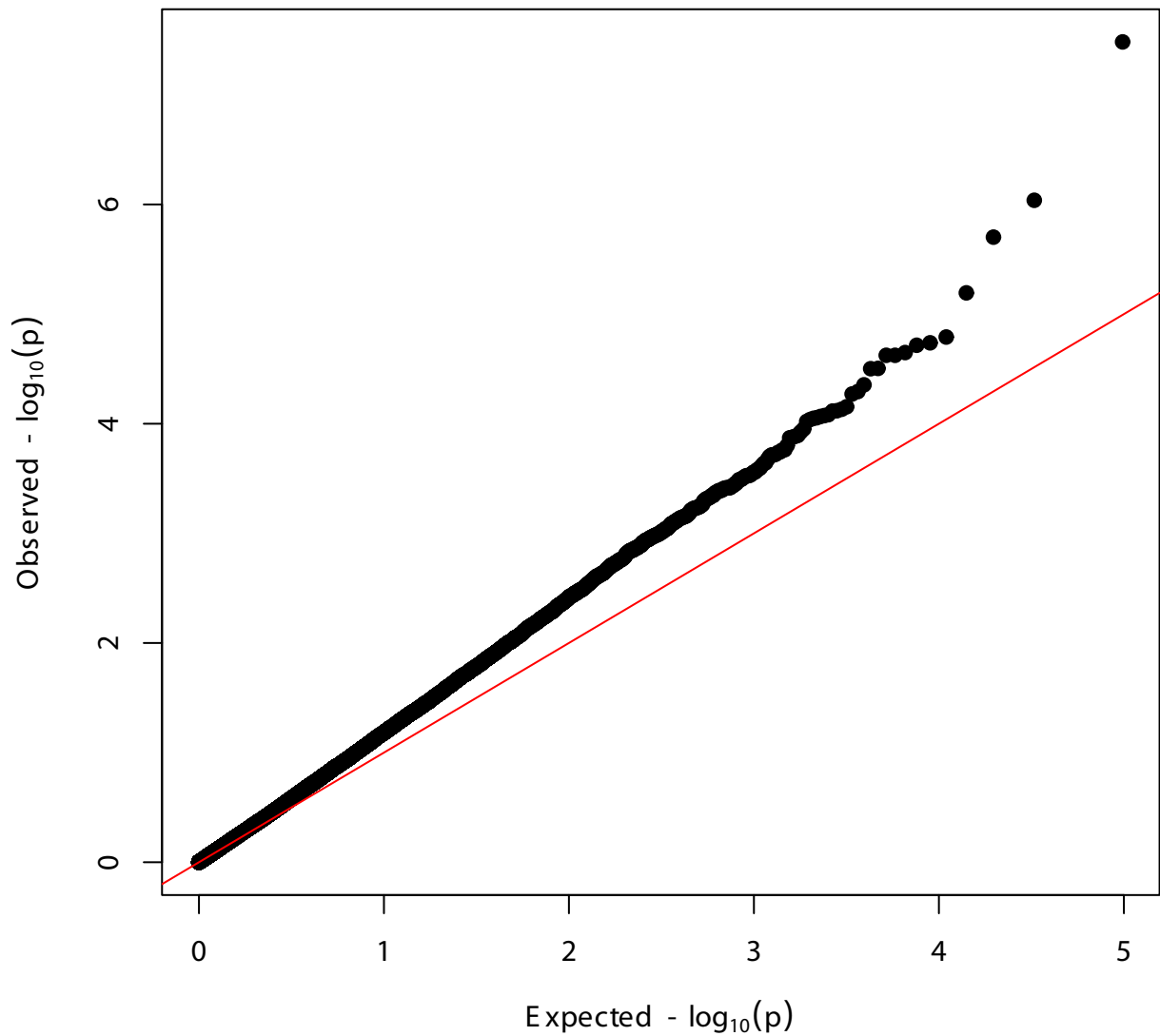

Supplement: Figure S3 — Quantile-Quantile plot for odds of infection. Quantile-quantile plots from association with serological status, where the red line shows the expected distribution. Representative data from the all-breeds, additive mode of inheritance analysis are shown. The results show deviation from the expected distribution indicating population stratification by factors unaccounted in the analytic model, which could include frequencies of underlying mutations for susceptibility loci that differ between seropositive and seronegative individuals. (PDF) [file pone.0047829.s003.pdf]

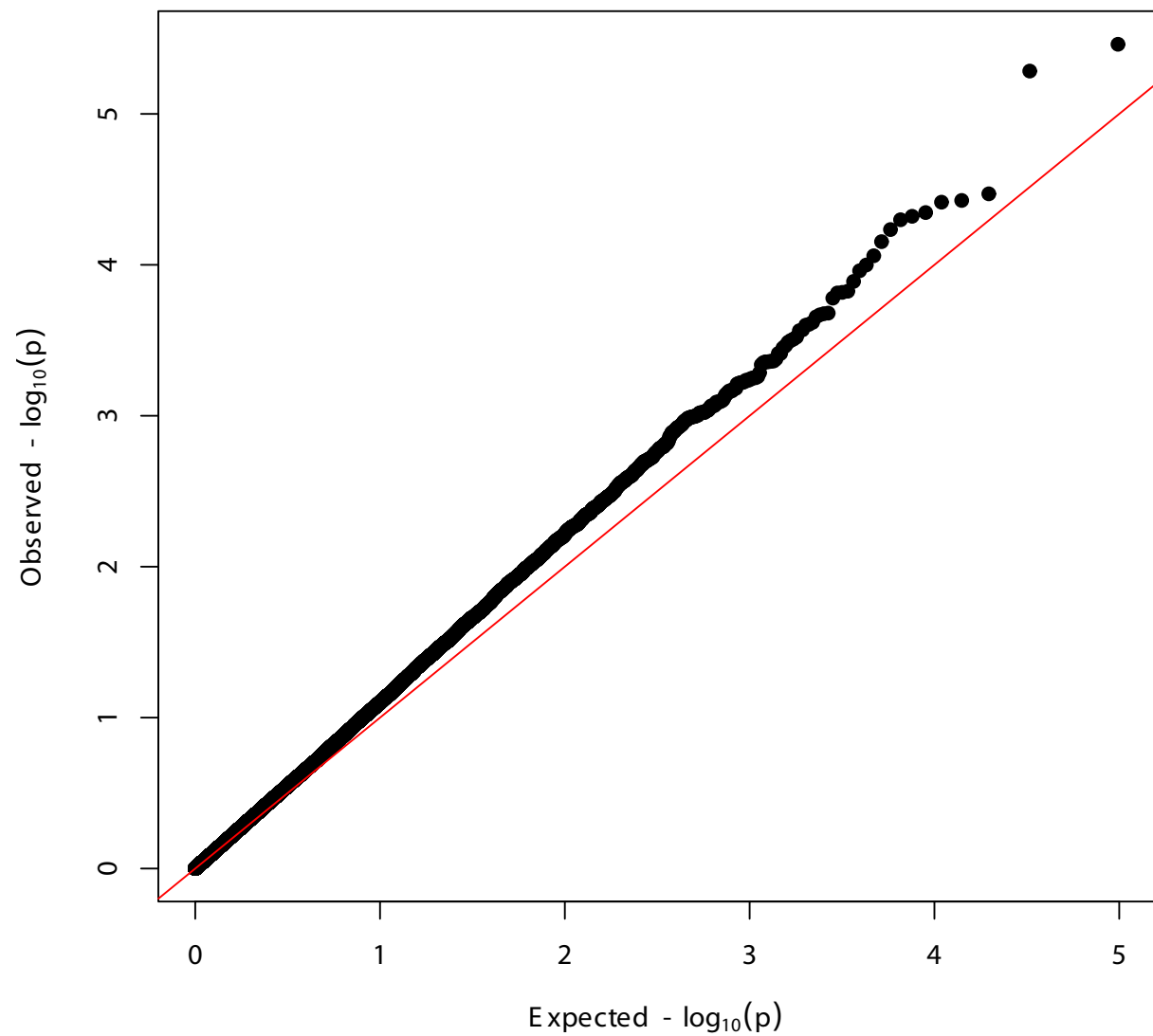

Supplement: Figure S4 — Quantile-Quantile plot for odds of infection conditioned on TMEM154 risk status. A second analysis conditioned on TMEM154 risk status shows an observed distribution closer to expected than the primary analysis, but still does not account for the majority of apparent population stratification. (PDF) [file pone.0047829.s004.pdf]

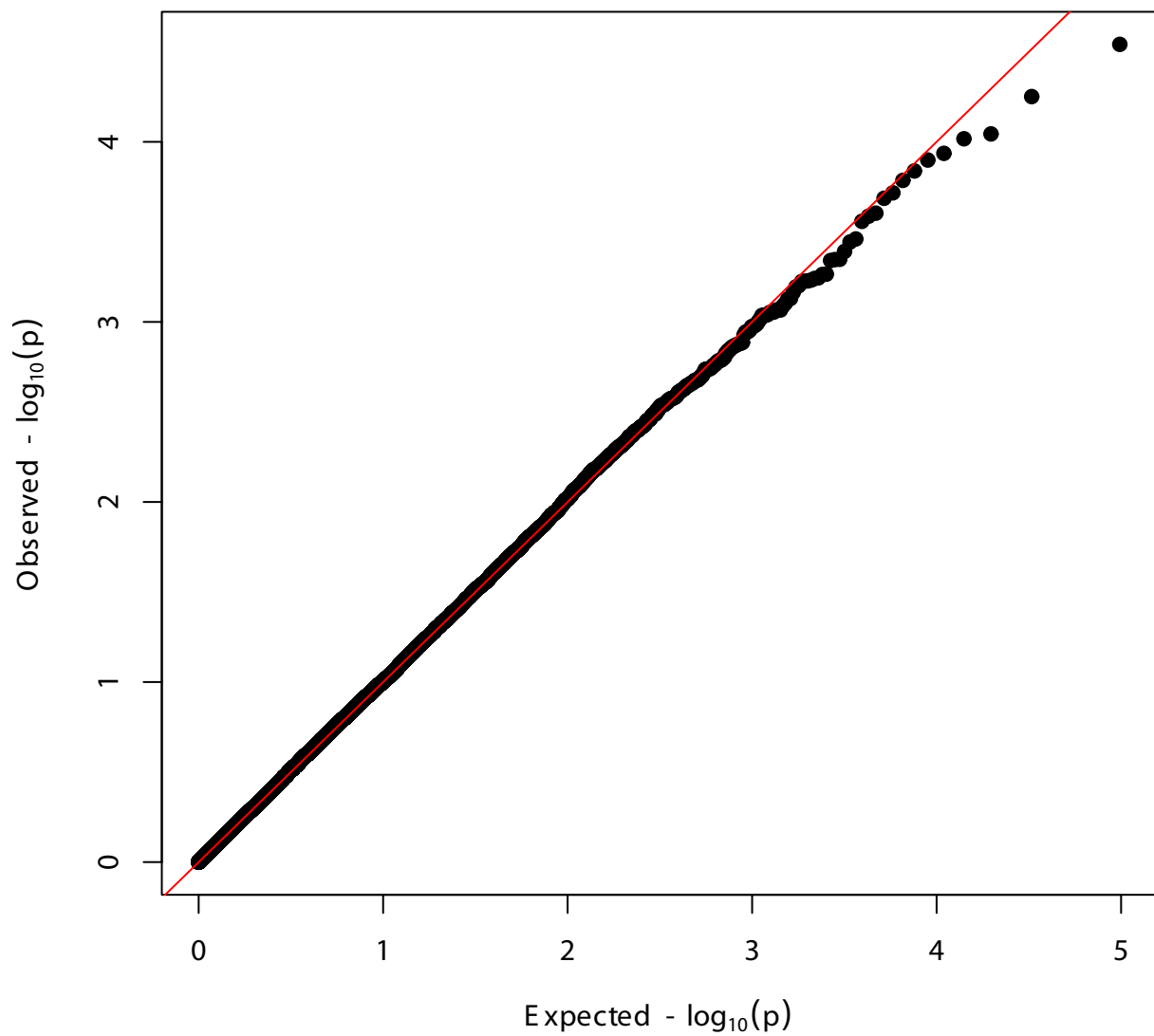

Supplement: Figure S5 — Quantile-Quantile plot for odds of infection conditioned on Table 1 SNP. A third analysis conditioned on all the SNP in Table 1 shows a distribution much closer to expected, demonstrating that host genetic factors tracked by these SNP account for the majority of apparent population stratification. (PDF) [file pone.0047829.s005.pdf]

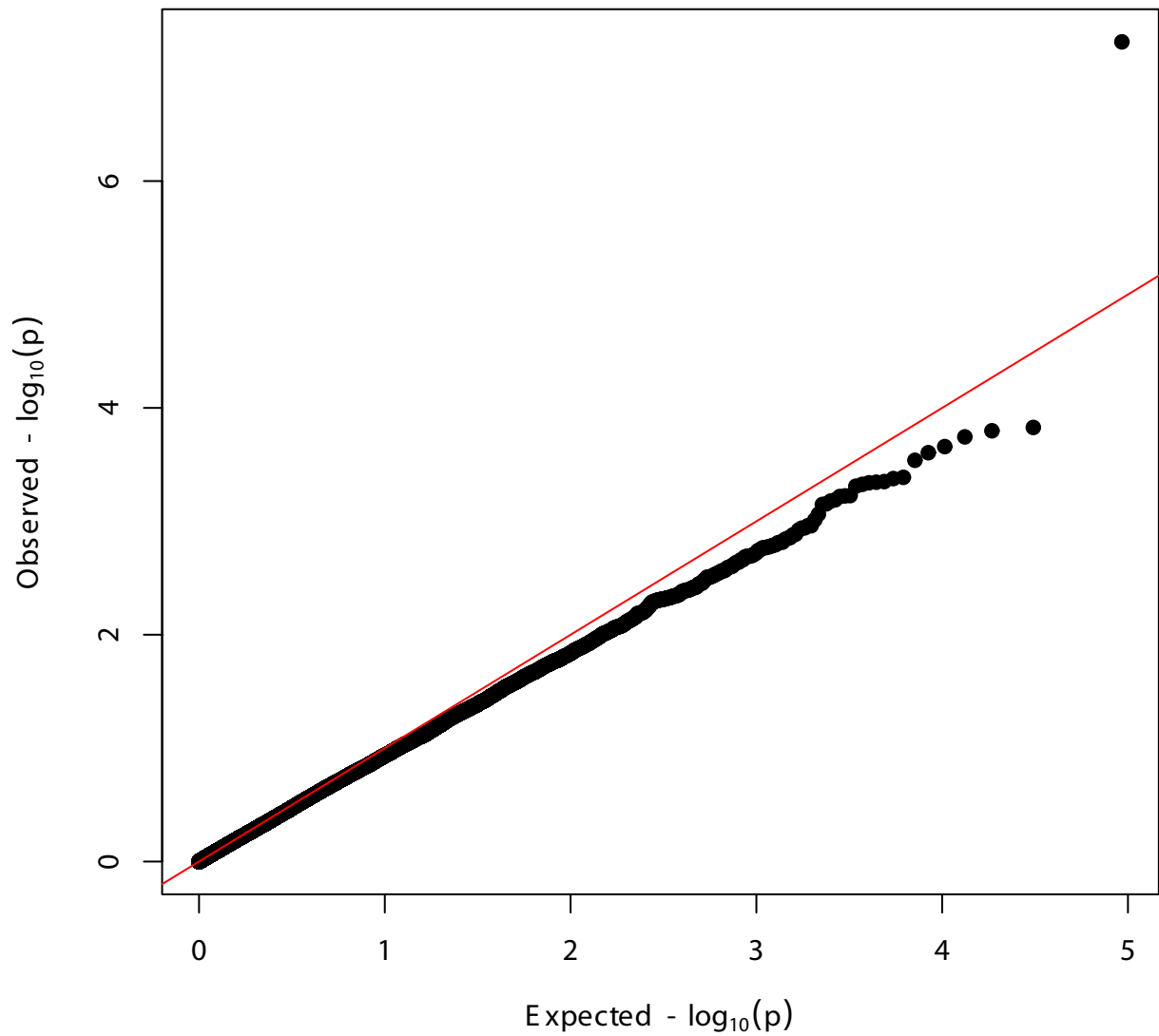

Supplement: Figure S6 — Quantile-Quantile plot for control of viral replication. Quantile-quantile plot from association with proviral concentration, where the red line shows the expected distribution. Representative data from the Rambouillet, dominant mode of inheritance analysis are shown. The results show deviation from the expected distribution indicating population stratification by factors unaccounted in the analytic model, which could include frequencies of underlying mutations for susceptibility loci that differ by proviral concentration. (PDF) [file pone.0047829.s006.pdf]

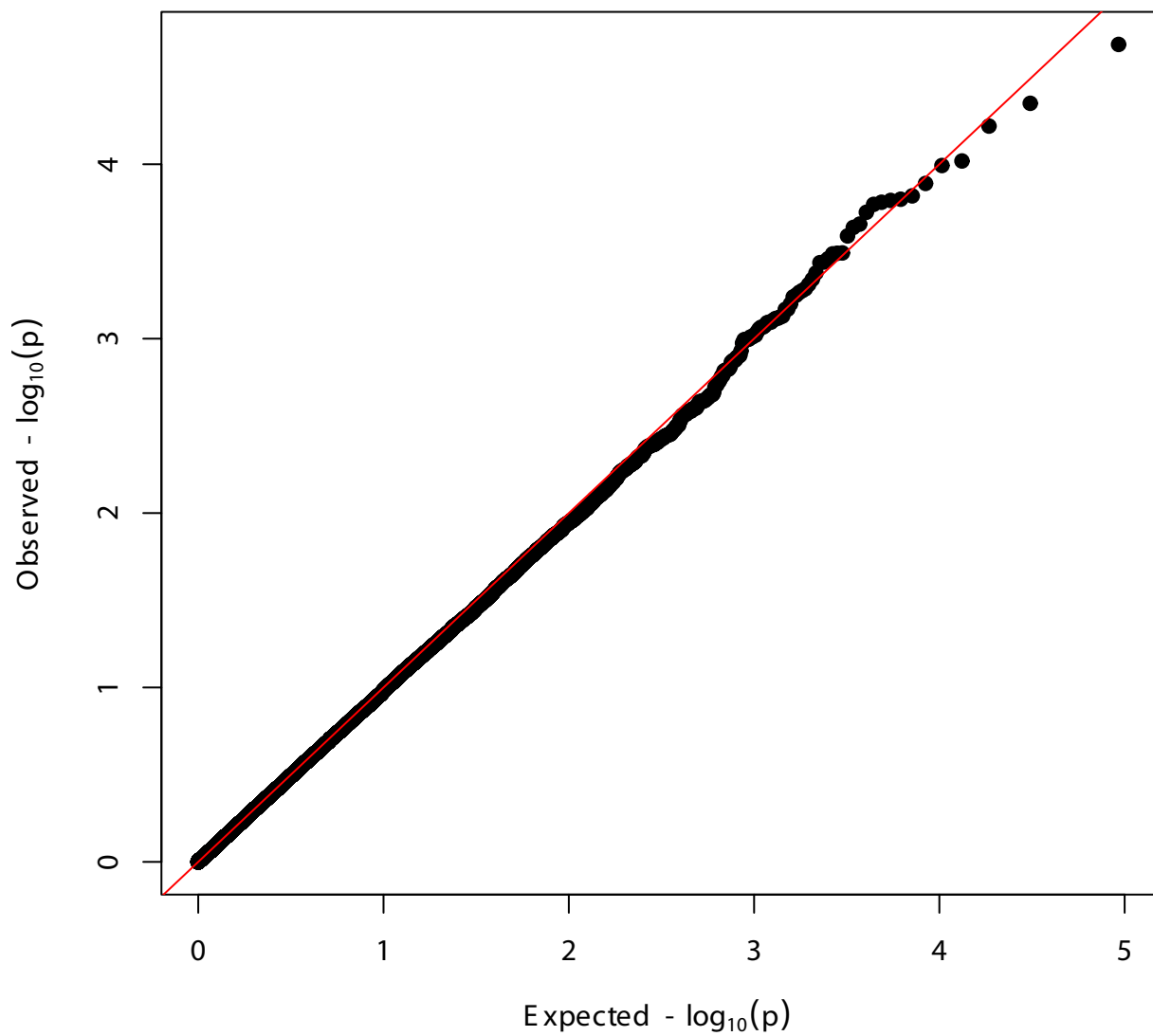

Supplement: Figure S7 — Quantile-Quantile plot for control of viral replication conditioned on Table 3 SNP. A second analysis was performed by conditioning on all the SNP in Table 3, minus close equivalents on the same chromosome (r2>0.8; removed to prevent inestimable multicollinearity) for which only the best P-value SNP was retained from each pair. This analysis shows a distribution much closer to expected, demonstrating that host genetic factors tracked by these SNP account for the majority of apparent population stratification. (PDF) [file pone.0047829.s007.pdf]
