# Supplementary material for: Genome-Wide Association Identifies Multiple Genomic Regions Associated with Susceptibility to and Control of Ovine Lentivirus
Source: PLoS One. 2012 Oct 17;7(10):e47829. doi: 10.1371/journal.pone.0047829 (PMC3474742; doi:10.1371/journal.pone.0047829)
Supplement: Table S1 — Adjusted genotypic mean proviral concentrations for Table 3 SNP. Mean proviral concentrations by genotype, adjusted for age and breed. (DOC) [file pone.0047829.s008.doc]

**Table S1 – Adjusted genotypic mean proviral concentrations for Table 3 SNP**

| *SNP* | *Animal Set* | *Allele 1* | *Allele 2* | *Adjusted Mean Log_10_ Proviral Concentration: Homozygote 1* | *Adjusted Mean Log_10_ Proviral Concentration: Heterozygote* | *Adjusted Mean Log_10_ Proviral Concentration: Homozygote 2* | *Nominal P-value* | *Empirical*  *P-value* | *Genotypic Log_10_ Conc. Diff.* | *Genes within 100 Kb on either side* |  |
| --- | --- | --- | --- | --- | --- | --- | --- | --- | --- | --- | --- |
| DU231007_156 | Polypay | C | T | 1.94 | 2.04 | 2.71 | 3.5x10^-6^ | § | 0.78 | *PAX8*, *IGK* |  |
| OAR3_144283427 | Polypay | A | T | 1.67 | 2.52 | 2.03 | 2.0x10^-6^ | § | 0.84 | *SLC11A2** |  |
| OAR3_144414855 | Polypay | A | G | 2.03 | 2.52 | 1.67 | 2.0x10^-6^ | § | 0.84 | *SLC11A2*** |  |
| s27054 | Polypay | A | G | 2.88 | 2.04 | 2.09 | 1.3x10^-6^ | 0.047 | 0.84 | *C19orf42***, *TMEM38A**, *NWD1*, *MED26*, *SLC35E1*, *CHERP* |  |
| OAR9_10735564 | Polypay | C | T | 2.57 | 2.46 | 1.81 | 1.6x10^-6^ | 0.073 | 0.76 | - |  |
| OAR9_10749779 | Polypay | A | G | 2.76 | 2.46 | 1.84 | 1.5x10^-6^ | 0.069 | 0.92 | - |  |
| s48118 | Polypay | C | T | 1.85 | 2.47 | 2.43 | 9.4x10^-6^ | § | 0.62 | *BAI1***, *LOC529919**, *ARC*, *RPL38* |  |
| OAR13_56607666 | All | A | G | 1.74 | 2.03 | 2.32 | 4.3x10^-6^ | § | 0.58 | *TGM6** |  |
| OAR18_5646940 | Polypay | C | T | 2.56 | 1.87 | 2.53 | 2.5x10^-6^ | § | 0.68 | *MEF2A*** |  |
| OAR18_5701234 | Polypay | C | T | 2.56 | 1.88 | 2.54 | 2.6x10^-6^ | § | 0.68 | *MEF2A*** |  |
| s65956 | Rambouillet | A | G | 2.40 | 1.61 | 1.51 | 5.9x10^-8^ | 0.001 | 0.89 | *ZNF192**, *ZSCAN16**, *ZNF165**, *ZNF389** |  |
| OAR22_43742889 | Polypay | A | G | 3.39 | 2.69 | 2.07 | 9.4x10^-6^ | § | 1.32 | *INPP5F***, *MCMBP*, *BAG3* |  |
| OAR23_40410527 | All | C | T | 1.87 | 2.24 | 2.53 | 1.5x10^-6^ | 0.092 | 0.66 | *DLGAP1*** |  |

^§^: P>0.15

**: SNP located within gene

*****: SNP located within 35 Kb of gene
