# Supplementary material for: Genome-Wide Association Identifies Multiple Genomic Regions Associated with Susceptibility to and Control of Ovine Lentivirus
Source: PLoS One. 2012 Oct 17;7(10):e47829. doi: 10.1371/journal.pone.0047829 (PMC3474742; doi:10.1371/journal.pone.0047829)
Supplement: Table S2 — Genomic regions from Columbia breed associated with proviral concentration of ovine lentivirus. Genomic regions from Columbia breed associated with proviral concentration of ovine lentivirus. (DOC) [file pone.0047829.s009.doc]

## Table S1 - Genomic regions from Columbia breed associated with control of ovine lentivirus replication

| *SNP* | *Chr* | *Position (bp)* | *Animal Set* | *Best fitting model* | *Allele 1* | *Allele 2* | | *Adjusted Mean Log_10_ Proviral Conc.: Homozyg 1* | *Adjusted Mean Log_10_ Proviral Conc.: Heterozyg* | *Adjusted Mean Log_10_ Proviral Conc.: Homozyg 2* | *Nominal P-value* | *Empirical*  *P-value* | *Genotypic Log_10_ Conc. Diff.* | *Genes within 100 Kb on either side* |
| --- | --- | --- | --- | --- | --- | --- | --- | --- | --- | --- | --- | --- | --- | --- |
| s23946^a^ | 3 | 101,585,304 | Columbia | genotypic | A | | C | 3.54 | 2.46 | 1.89 | 1.9x10^-7^ | 0.032 | 1.66 | *AFF3**, *REV1* |
| OAR3_108176200 | 3 | 101,534,582 | Columbia | genotypic | C | | T | 1.86 | 2.63 | 3.48 | 2.9x10^-6^ | § | 1.63 | *AFF3*** |
| OAR5_93280925 | 5 | 85,630,981 | Columbia | genotypic | C | | T | 3.21 | 2.08 | 1.90 | 1.6x10^-6^ | § | 1.31 | *TMEM161B** |
| OAR5_94009731 | 5 | 86,343,222 | Columbia | genotypic | A | | C | 2.22 | 1.91 | 3.05 | 4.8x10^-6^ | § | 1.14 | *MEF2C* |
| s27979 | 8 | 28,820,456 | Columbia | genotypic | A | | G | 2.96 | 1.79 | 2.40 | 7.8x10^-8^ | § | 1.17 | *FOXO3*** |
| OAR13_26084492 | 13 | 23,579,320 | Columbia | genotypic | C | | T | 3.23 | 1.87 | 2.61 | 8.7x10^-6^ | § | 1.36 | - |
| OAR15_78598295 | 15 | 72,758,328 | Columbia | genotypic | A | | G | 2.79 | 1.91 | 1.11 | 2.3x10^-6^ | § | 1.67 | *EXT2***, *ALX4** |
| s31340 | 15 | 72,774,077 | Columbia | genotypic | A | | C | 3.01 | 1.99 | 1.59 | 2.1x10^-7^ | § | 1.42 | *EXT2***, *ALX4** |
| OAR16_54540482 | 16 | 50,293,617 | Columbia | genotypic | A | | G | 2.96 | 2.48 | 1.58 | 6.5x10^-6^ | § | 1.38 | - |
| OAR21_623459 | 21 | 516,001 | Columbia | genotypic | A | | G | 3.55 | 1.95 | 2.40 | 8.8x10^-6^ | § | 1.60 | *MED17***, *C11orf54*, *TAF1D* |

## ^a^: This marker did not validate in large-scale testing done in conjunction with another study [14]. Specifically, there was evidence of a nearby SNP that could interfere with base-calling by shifting X and Y signal. However, examination of X and Y data revealed clear overall genotype clustering in these Columbia animals. Further, results from nearby SNP OAR3_108176200 confirm association of this genomic region.

## ^§^: P>0.15

## **: SNP located within gene

*****: SNP located within 35 Kb of gene
